# Supplementary material for: FOXD1 Is a Transcription Factor Important for Uveal Melanocyte Development and Associated with High-Risk Uveal Melanoma
Source: Cancers (Basel). 2022 Jul 28;14(15):3668. doi: 10.3390/cancers14153668 (PMC9367502; doi:10.3390/cancers14153668)
Supplement: Supplementary file 1 [file UM in silico FOXD1 supplementary rebuttal v5.pdf › UM in silico FOXD1 supplementary rebuttal v5.pdf]

cancers

Supplementary material

FOXD1 Is a Transcription Factor Important for Uveal Melanocyte Development and Associated with High-Risk Uveal Melanoma

Quincy C.C. van den Bosch, Josephine Q.N. Nguyen, Tom Brands, Thierry P.P. van den Bosch, Robert M. Verdijk[3,4], Dion Paridaens, Nicole C. Naus, Annelies de Klein, Emine Kiliç Erwin Brosens, on behalf of the Rotterdam Ocular Melanoma Study group

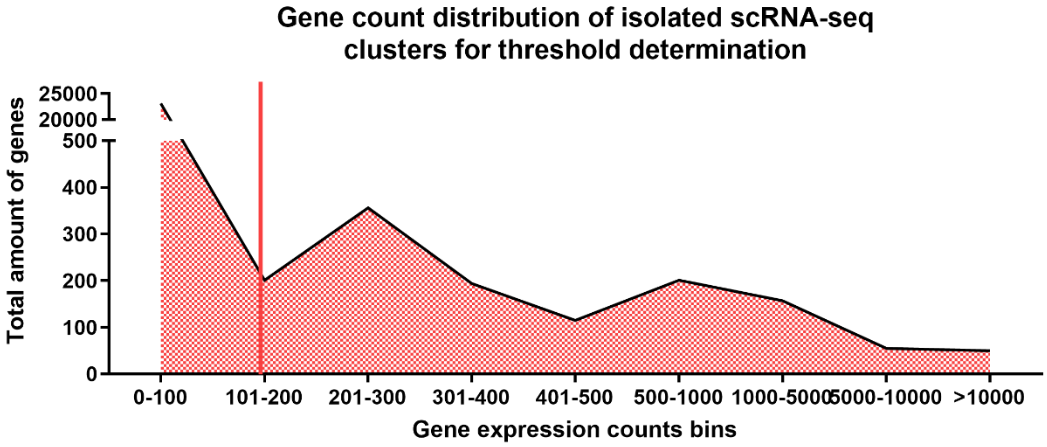

Figure S1. Determination of the read counts at 100 based on overall gene counts distribution.

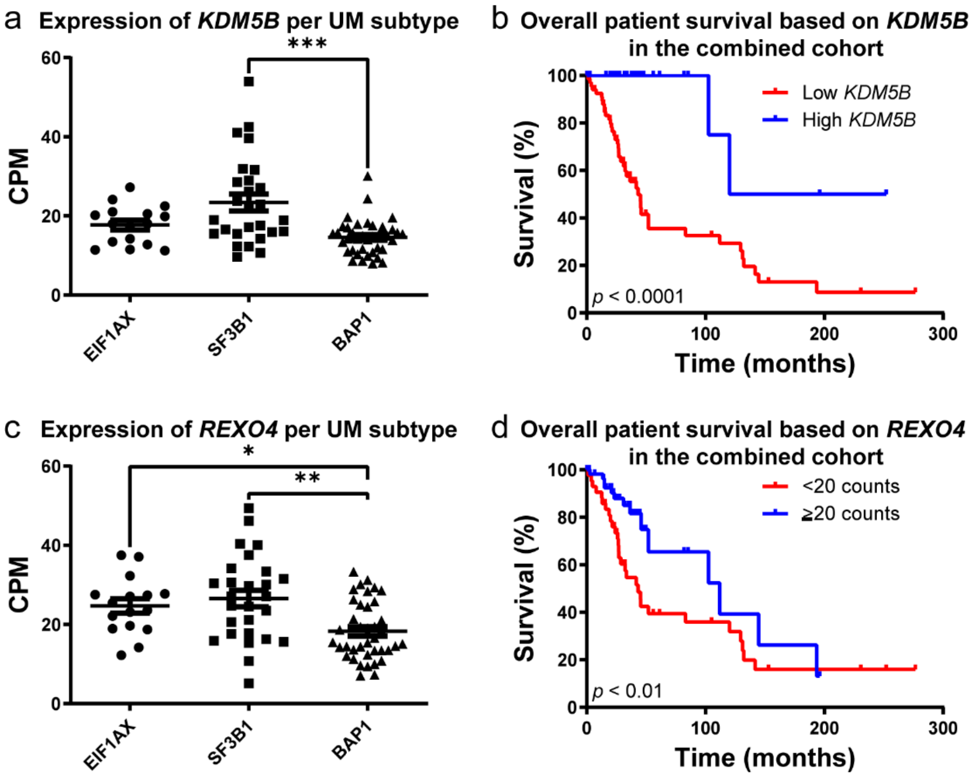

Figure S2. Expression per UM mutational subtype and survival plot of *KDM5B* (**a**,**b**) and *REXO4* (**c**,**d**) in the combined cohort. A low expression of *KDM5B* is correlated with poor prognosis ($p < 0.0001$) and the expression is significantly low in the *BAP1*-mutated group. *, $p < 0.05$; **, $p < 0.01$ ***, $p < 0.001$. A low expression of *REXO4* is also correlated with poor prognosis ($p < 0.01$) and the expression is significantly low in the *BAP1*-mutated group.

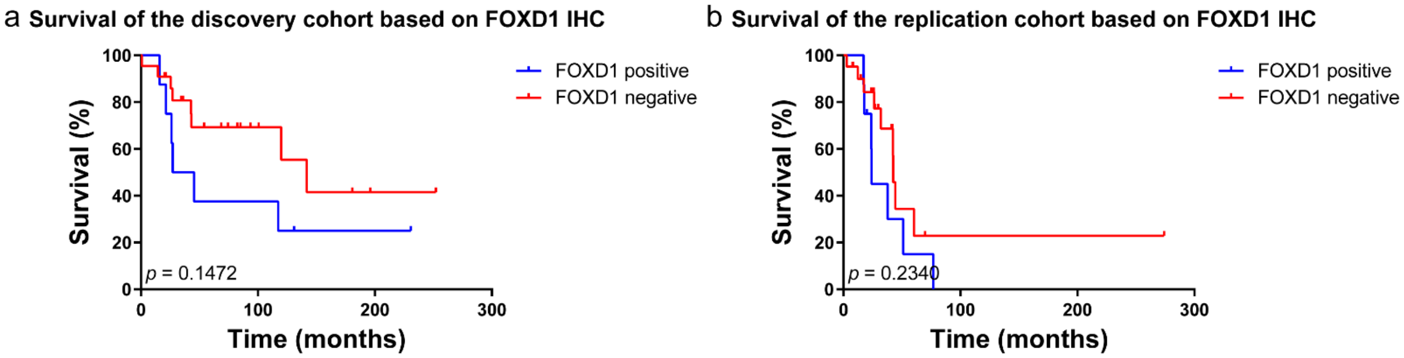

Figure S3. The survival plot of FOXD1 expression on IHC in the **a)** discovery cohort and the **b)** replication cohort. In both cohorts, no significant differences in survival were assessed based on FOXD1 protein expression (discovery cohort, $p = 0.1472$; replication cohort, $p = 0.2340$).

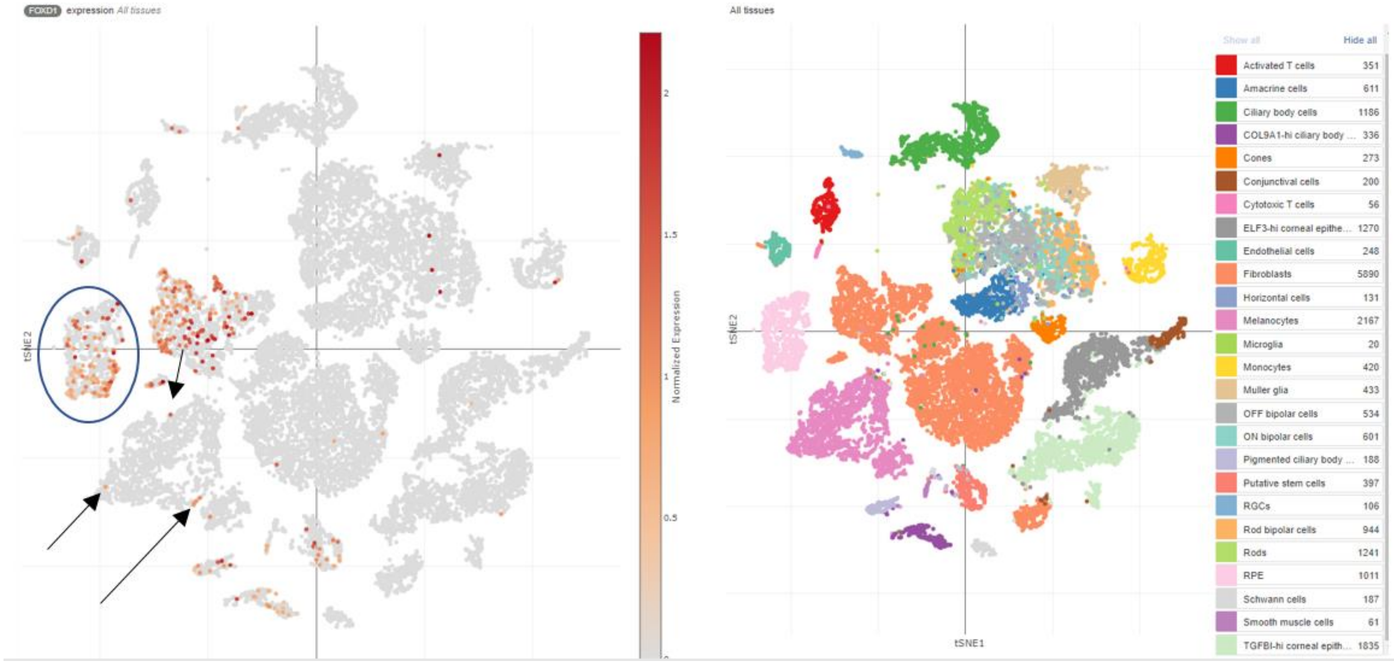

Figure S4. Uniform manifold approximation and projection (UMAP) of ocular compartments (Gautam et al., Nat Commun, 2021) using the single-cell explorer of the Broad Institute. Black arrows show expression of *FOXD1* in ocular melanocytes. Blue circle highlights RPE cells which also show expression of *FOXD1*.

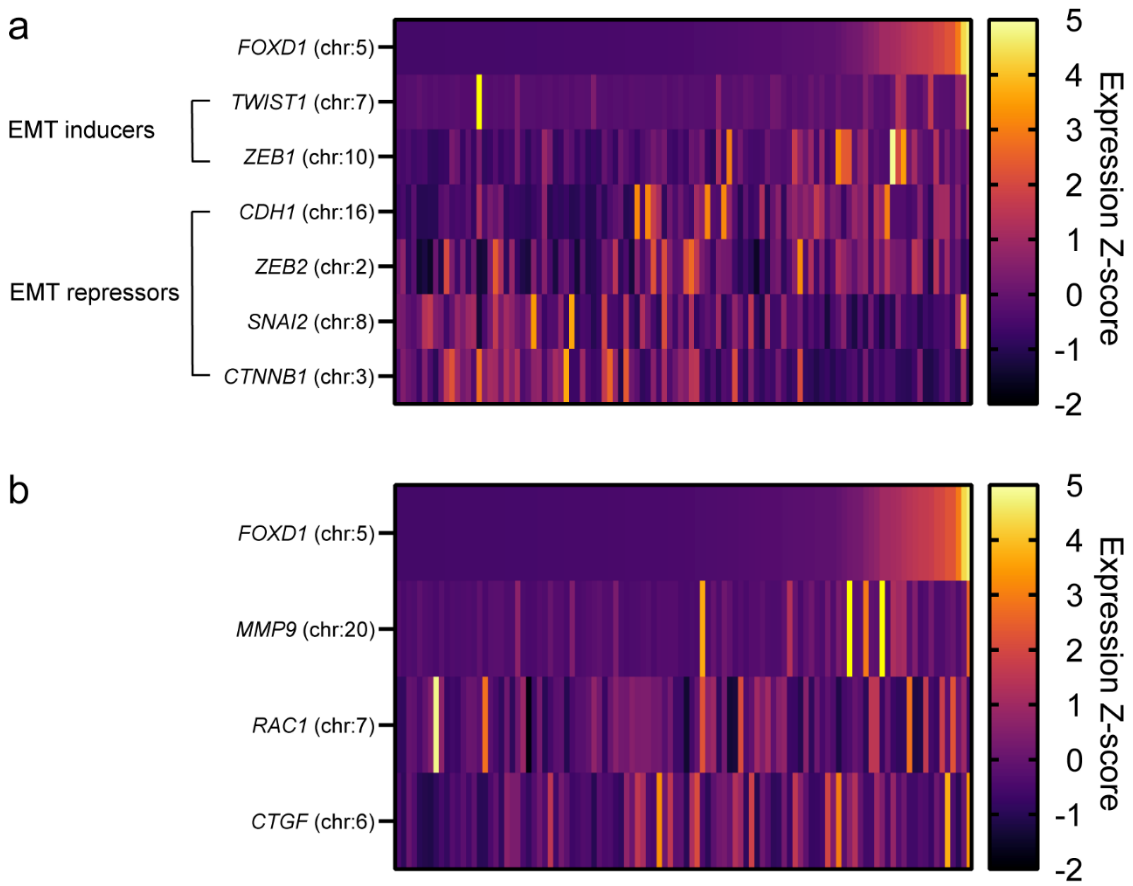

Figure S5. a) Heatmap using Z-score of *FOXD1* next to EMT inducers and repressors on UM samples. The pattern of *FOXD1* does not seem to correlate to an EMT related pattern. **b)** Heatmap using Z-score of *FOXD1* next to EMT genes described in cutaneous melanoma.
